# Validation of venous thromboembolism diagnoses in patients receiving rivaroxaban or warfarin in The Health Improvement Network

**DOI:** 10.1002/pds.5146

**Published:** 2020-10-12

**Authors:** Ana Ruigómez, Gunnar Brobert, Pareen Vora, Luis A. García Rodríguez

**Affiliations:** ^1^ Spanish Centre for Pharmacoepidemiologic Research (CEIFE) Madrid Spain; ^2^ Bayer AB Stockholm Sweden; ^3^ Bayer AG Berlin Germany

**Keywords:** venous thromboembolism, venous thrombosis, pulmonary embolism, database, incidence, anticoagulants

## Abstract

**Purpose:**

To describe the effect that validation of venous thromboembolism (VTE) coded entries in the health improvement network (THIN) has on incidence rates of VTE among a cohort of rivaroxaban/warfarin users.

**Methods:**

Among 36 701 individuals with a first prescription for rivaroxaban/warfarin between 2012 and 2015, we performed a two‐step VTE case identification process followed by a two‐step case validation process involving manual review of patient records. A valid case required a coded entry for VTE at some point after their first rivaroxaban/warfarin prescription with evidence of referral/hospitalization either as a coded entry or entered as free text. Positive predictive values (PPVs) with 95% confidence intervals (CIs) were calculated using validated cases as the gold standard. Incidence rates were calculated per 1000 person‐years with 95% CIs.

**Results:**

We identified 2166 patients with a coded entry of VTE after their initial rivaroxaban/warfarin prescription; incidence rate of 45.31 per 1000 person‐years (95% CI: 43.49‐47.22). After manual review of patient records including the free text, there were 712 incident VTE cases; incidence rate of 14.90 per 1000 person‐years (95% CI: 13.85‐16.02). The PPV for coded entries of VTE alone was 32.9%, and the PPV for coded entries of VTE with a coded entry of referral/hospitalization was 39.8%; this increased to 69.6% after manual review of coded clinical entries in patient records.

**Conclusions:**

Among rivaroxaban/warfarin users in THIN, valid VTE case identification requires manual review of patient records including the free text to prevent outcome misclassification and substantial overestimation of VTE incidence rates.

**Key Points**

- Use of only coded clinical information in THIN database is insufficient to accurately identify incident cases of major venous thromboembolism (VTE).
- Manual review of patient records substantially increases the validity of VTE cases identified through algorithmic searches for coded diagnoses.
- The free text comments in THIN commonly provide clinical information important for valid case identification.
- A manual review process including scrutiny of the free text comments is a valid method to identify cases of VTE and avoid misclassification, especially to reduce false positives.
- Without manual review of free‐text comments in THIN, incidence rates of VTE will be substantially overestimated.

## 1 INTRODUCTION

Rivaroxaban is one of several non‐vitamin K antagonist oral anticoagulants (NOACs) licensed in the United Kingdom for the treatment of venous thromboembolism (VTE), prophylaxis of VTE after knee/hip surgery, and prophylaxis of recurrent VTE.^1^ Since 2016, rivaroxaban has been the most common oral anticoagulant prescribed to patients in England with incident VTE.^2^ Approval of rivaroxaban for VTE indications was based on data from randomized controlled trials (RCTs)^3^, ^4^, ^5^ with strict inclusion/exclusion criteria. Thus far, data on the effectiveness of rivaroxaban for VTE indications among the broad spectrum of patients receiving the drug in routine clinical practice have come from patient registry^6^, ^7^ or claims database studies,^8^ or observational field studies.^9^, ^10^

Databases of electronic health records (EHRs) are other appropriate sources to efficiently conduct rivaroxaban effectiveness studies. One such database—The Health Improvement Network (THIN)—has been used extensively for pharmacoepidemiological research. It holds the pseudo‐anonymized primary care EHRs of approximately 6% of the UK population,^11^ who are broadly representative of the UK demographic.^12^ As of September 3, 2017, 1 million patients were actively contributing patient data through their THIN participating practice.^13^ Data are recorded by the primary care practitioner (PCP) and other practice staff using Vision software during or after each consultation, or retrospectively after receiving information from secondary care via postal letter or email. Diagnoses are entered via Read codes, the clinical classification system used by the UK's National Health Service.^14^ After entering a Read code, a comment box opens in which the PCP can freely enter associated details, such as a referral to hospital, symptoms, or factors relating to the diagnostic work‐up—these can also be entered in part via Read codes if an appropriate code exists.

It is recommended that outcome identification using primary care databases such as THIN involves supportive evidence to validate the recorded diagnosis and avoid misclassification.^15^, ^16^ False negatives may arise through searches for Read codes for clinical entries supporting the diagnosis (eg, for a code for a hospitalization) during an overly restricted time interval, or if supporting information is recorded in the free text and these data are not accessed and reviewed. Conversely, false positives will occur if the free text refers to a previous/historical episode or confirms the absence of the event—an important factor to consider due to importance of achieving high case specificity in drug effectiveness/safety studies. However, access to free text comments in THIN requires an additional cost, and scrutiny of the comments is labor intensive. This study explored a stepwise validation process of VTE Read code entries in THIN among a cohort of oral anticoagulant users (new users of warfarin or rivaroxaban). The primary objective was to validate cases of VTE through a process involving review of coded clinical entries and free text comments. The secondary objective was to describe the effect that inclusion of a validation step involving the review of free text comments (vs Read code entries only) has on incidence rates of major VTE events among this cohort of patients.

## 2 METHODS

### 2.1 Study cohort and follow‐up

Details of the study cohort including the age and sex distribution have been published previously.^17^ Briefly, we included 36 701 individuals in THIN aged between 2 and 89 years with a first prescription for rivaroxaban or warfarin between January 1, 2012 and May 31, 2015. Individuals were followed‐up from the date of their first rivaroxaban/warfarin prescription (start date) to identify the first recorded VTE after this first prescription (individuals may therefore have had a VTE before the start of follow‐up). End of follow‐up was the earliest of the following: a Read code indicative/suggestive of deep vein thrombosis (DVT) or pulmonary embolism (PE) (see Table S1 for the code list), death, the date of the last data collection from their practice, or the end of the study period (May 31, 2015).

### 2.2 Case identification

Our operational definition of the first VTE event recorded during follow‐up was a VTE event that led to a referral either to a specialist or to hospitalization, or was recorded in the primary care record as the cause of death. We did not restrict to cases with evidence of hospital admission because a previous VTE validation study in the Clinical Practice Research Datalink (CPRD, formerly the General Practice Research Database—a highly similar database to THIN), found that approximately 20% of VTE cases confirmed by the PCP via paper questionnaires did not have a database entry indicative of hospital admission for the event.^18^

**FIGURE 1:**
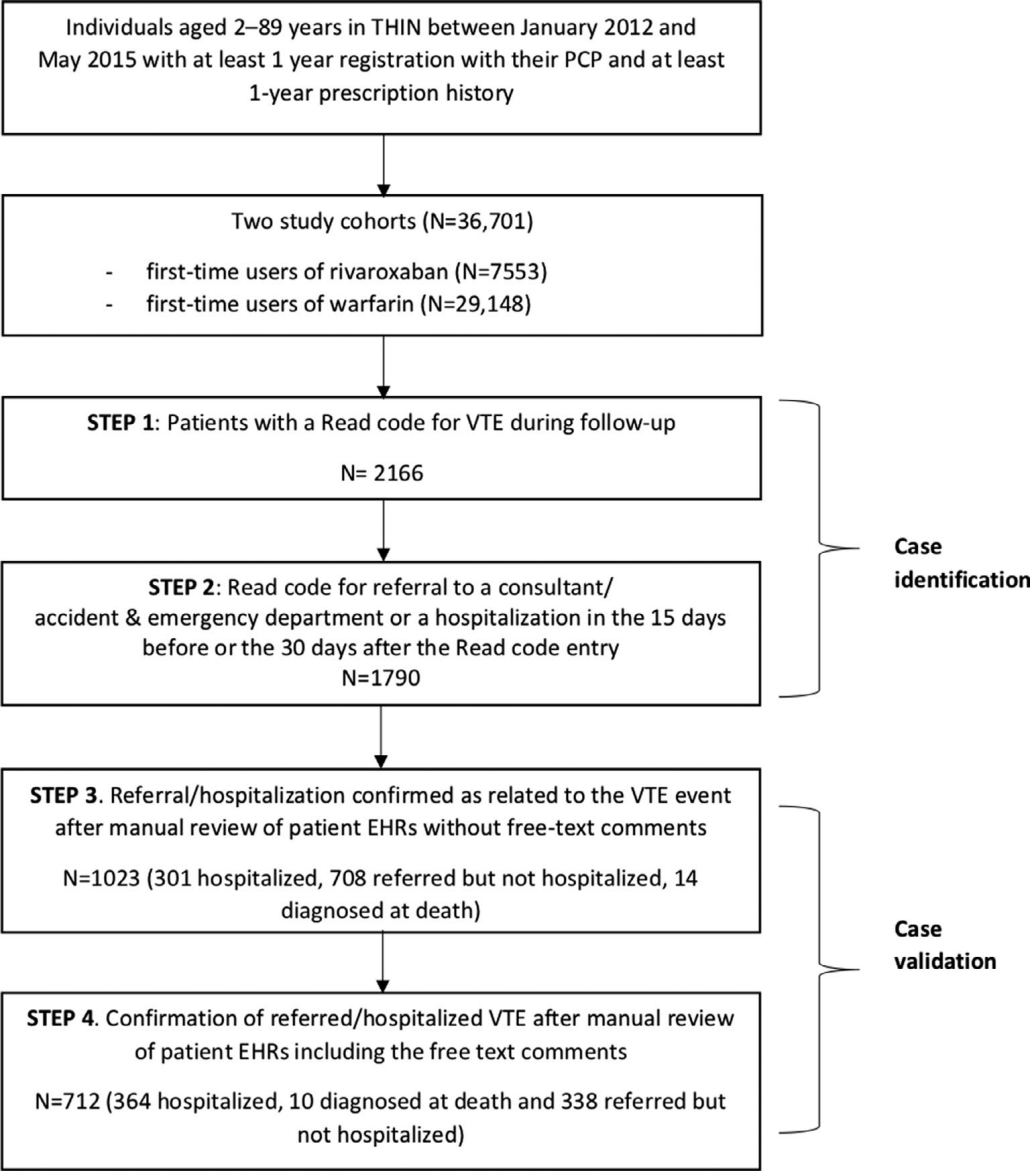
Flow diagram depicting the VTE case identification and validation process. EHR, electronic health records; PCP, primary care practitioner; PE, pulmonary embolism; THIN, The Health Improvement Network; VTE, venous thromboembolism

As shown in Figure 1, VTE case identification and validation involved a four‐step sequential process. Step 1 of the case identification process involved an automated computer search to identify patients with a Read code for VTE during follow‐up. In step 2, we performed automated computer searches among the EHRs of patients identified in step 1 to identify those with a specific entry or Read code for a referral to secondary care and/or a hospitalization in the 15 days before the VTE record or in the 30 days after. This time frame was applied to maximize the sensitivity of our case definition due to the fact that referrals/hospitalizations are not always recorded on the same day as a clinical diagnosis in a patient's EHR. All patients identified during this step were considered to be potential cases of VTE.

### 2.3 Validation of VTE

In Step 3, we manually reviewed the coded entries in the primary care record of potential VTE cases identified in the previous step to confirm the diagnosis and that the referral/hospitalization was related to the VTE event. For potential cases retained after this step 3, we then requested and accessed free‐text comments in the patients' primary care record for further manual review and final confirmation of the VTE event. To undertake this review efficiently we only accessed free text comments entered in the 15 days either side of the event, as well as all those specifically attached to an entry of DVT/PE or to any entry of hospitalization or referral in the 16 to 180 days after the event. These comments often contain information on referrals and details from hospital discharge letters describing the clinical evaluation and tests performed (eg, radiology tests and reports), as well as information from death certificates. A first manual review of these free‐text comments was performed independently by one researcher (AR) to confirm VTE case status and to establish whether the text referred to a current event or to a previous event. Subsequently, potential cases were grouped according to three main characteristics: (a) the interval between the date of first rivaroxaban/warfarin prescription and the date of the recorded VTE, (b) the indication for the anticoagulation (VTE or other), and (c) whether it was a recorded hospitalization or a referral to a specialist that led to the case being confirmed. For cases where the status was not clear after this review, a second independent review was performed by another researcher (LAGR) and consensus on case status was reached through discussion.

### 2.4 Statistical analysis

For cases identified in each step, we calculated the incidence rate of VTE per 1000 person‐years with 95% confidence interval (CI) as the number of first VTE cases identified during follow‐up divided by the corresponding observed person‐years. The incidence rate of final confirmed hospitalized/referred VTE cases was also calculated according to referral/hospitalization case status. We calculated the positive predictive value (PPV) of each validation step, as the ratio of the number of participants with a confirmed diagnosis after final confirmation (step 4) to number of patients with a VTE diagnosis identified after each step, and expressed this as a percentage with 95% CIs. As a post‐hoc analysis, stratified analyses were performed calculating the confirmation rate according to age at the start of follow‐up (<60 years, 60‐69 years, and 70‐89 years), sex, indication for the first rivaroxaban/warfarin prescription, VTE diagnosis (ie, DVT or PE), time between the first rivaroxaban/warfarin prescription and VTE event during follow‐up, history of VTE (ie, before the start date), hospitalization/death or referral related to the VTE diagnosis, and specific VTE Read code entry.

## 3 RESULTS

### 3.1 Identified and confirmed cases

During the automatic computer algorithm searches in steps 1 and 2, a total of 2166 patients were identified with a Read code for VTE, and 1790 (82.6%) of these had a Read code for a referral to a specialist and/or a hospitalization during the specified time period and were consequently deemed to be potential cases. A total of 1023 (57.2%) of these patients had the referral/hospitalization confirmed as being related to the VTE event after the first manual review validation process in step 3 (before accessing free text comments). Of these 1023 patients, 301 had the hospitalization confirmed as related to the VTE event, 708 had no confirmed hospitalization but were confirmed as having a referral to a specialist, and 14 patients had DVT recorded on the date of death. As shown in Figure 1, after step 3, less than half (47.2%) of the 2166 patients identified with a VTE Read code in step 1 were retained as potential major cases. After the final validation step, involving the second manual review of the patients' primary care record with the free text comments, the VTE diagnosis and the referral/hospitalization was confirmed as being relating to the VTE event in 712 patients; (69.6% of the 1023 potential patients reviewed in step 3). As shown in Table 1, among these 712 confirmed cases, 374 (52.5%) had been hospitalized or DVT was the cause of death, and 338 (47.5%) had been referred to specialist for DVT and not hospitalized. There were 311 patients who were not confirmed as having a major VTE event during this final validation step. Reasons for deeming these patients as non‐cases were either: the VTE Read code entry referred to a past event (178 patients), or the referral/hospitalization was related to another clinical condition (ie, not VTE; 133 patients).

**TABLE 1:** Validation summary: final confirmed cases after step 4 as a percentage of possible patients residing at step 3

	Case status after manual review with free‐text comments (step 4)	Confirmation rate (N confirmed/N revised at step 3 = 1023 × 100)
Confirmed cases (all)	712	69.6
Confirmed VTE with hospitalization (n = 364) or confirmed as cause of death (n = 10)	374	36.6
Confirmed VTE with referral to a specialist or a hospital accident and emergency department (ie, no hospital admission)	338	33.0
Non‐confirmed cases of VTE, n (%)	311	30.4
Referral/hospitalization was not confirmed as relating to the VTE event	133	13.0
Past event (ie, entry referred to a previous VTE event)	178	17.4

Abbreviation: VTE, venous thromboembolism. Data are n (%).

### 3.2 Positive predictive values and incidence rate of VTE

Incidence rates of VTE and the PPV of cases identified at each step of the case identification process, using cases confirmed from the final step (step 4) as the gold standard, are shown in Table 2. The PPV of Read code entries for VTE in the study cohort (ie, cases identified after step 1) was 32.9%, and the incidence rate was 45.31 per 1000 person‐years (95% CI: 43.49‐47.22). The PPV for coded entries of VTE plus a coded entry for a referral/hospitalization in the specified time frame (ie, cases identified after step 2) was 39.8%; this increased to 69.6% after manual review of patient records excluding the free‐text comments (after step 3). The incidence rate of major VTE (hospitalized/cause of death/or referred but not hospitalized cases) using the final 712 validated cases after manual final review of free‐text comments (ie, cases confirmed after step 4) was 14.90 per 1000 person‐years (95% CI: 13.85‐16.02), and only was 7.82 per 1000 person‐years (95% CI: 7.07‐8.65), when restricting to hospitalized/VTE as cause of death cases.

**TABLE 2:** Incidence rates of VTE, and PPV, of cases identified at each step of the case identification process using cases identified from step 4 as the gold standard

	Cases	Person‐years	Incidence rates per 1000 person‐years (95% CI)	Confirmed cases after Step 4, n	PPV (95% CI), %
**Step1:** VTE Read codes only	2166	47 800	45.31 (43.49‐47.22)	712	32.9 (30‐9‐34.9)
**Step 2:** VTE Read code plus referral/hospital Read code	1790	47 800	37.45 (35.78‐39.19)	712	39.8 (37.5‐42.1)
**Step 3:** Following manual review of patient EHRs without free‐text comments	1023	47 800	21.40 (20.14‐22.74)	712	69.6 (66.7‐72.3)
**Step 4:** Final major VTE definition^a^	712	47 800	14.90 (13.85‐16.02)	NA	NA
Hospital VTE	374	47 800	7.82 (7.07‐8.65)	NA	NA

Abbreviations: CI, confidence interval; DVT, deep vein thrombosis; NA, not applicable; PE, pulmonary embolism, PPV, positive predictive value.

^a^ Final VTE definition: a Read code for VTE, with a related referral or hospitalization confirmed after manual review of patient EHRs with the free‐text comments.

In stratified analyses, variation was seen in the confirmation rate across patient characteristics (Table 3). Of all potential VTE reviewed, there were more confirmed cases among males, older patients (aged ≥60 years), among those with a history of VTE (for recurrent events), and among those where the indication for the first rivaroxaban/warfarin prescription was for VTE (rather than another indication for example, atrial fibrillation). A higher confirmation rate was also seen among patients whose VTE event was a PE rather than a DVT, among patients hospitalized for their VTE, and when the time between the first rivaroxaban/warfarin prescription and the VTE event was >90 days. Two Read codes—G801.11 and G401.00—were responsible for identifying close to 85% of all confirmed VTE cases.

**TABLE 3:** Distribution of main characteristics considered during validation, and confirmation rate comparing with the previous validation step

	Not confirmed		Confirmed major VTE cases		Total revised with free text (Step 3)	Confirmation rate (95% CI), %
	N	%	N	%	N	(N confirmed/N revised)×100
**TOTAL**	**311**		**712**		**1023**	**69.6 (66.7**–**72.3)**
**Sex**						
Male	135	43.4	360	50.6	495	72.7 (68.6‐76.5)
Female	176	56.6	352	49.4	528	66.7 (62.5‐70.6)
**Age at start of follow‐up (years)**						
<60	116	37.3	242	34.0	358	67.6 (62.6‐72.2)
60‐69	63	20.3	151	21.2	214	70.6 (64.1‐76.3)
70‐89	132	42.4	319	44.8	451	70.7 (66.4‐74.7)
**Time between rivaroxaban/warfarin prescription and VTE (days)**						
1‐90	108	34.7	133	18.7	241	55.2 (48.9‐61.3)
>90	203	65.3	579	81.3	782	74.0 (70.9‐77.0)
**Rivaroxaban/warfarin indication**						
DVT/PE	210	67.5	526	73.9	736	71.5 (68.1‐74.6)
Other (eg, NVAF)	101	32.5	186	26.1	287	64.8 (59.1‐70.1)
**History of DVT/PE (before the start date)**						
No	116	37.3	193	27.1	309	62.5 (56.9‐67.7)
Yes	195	62.7	519	72.9	714	72.7 (69.3‐75.8)
**VTE diagnosis**						
DVT	199	64.0	418	58.7	617	67.7 (64.0‐71.3)
PE	112	36.0	294	41.3	406	72.4 (67.9‐76.5)
**VTE Read code** ^a^						
G801.11‐Deep vein thrombosis	94	30.2	329	46.2	423	77.8 (73.6‐81.5)
G401.00‐Pulmonary embolism	106	34.1	286	40.2	392	73.0 (68.4‐77.1)
G80.00‐Phleb/thrombophlebitis	62	19.9	27	3.8	89	30.3 (21.8‐40.5)
G801.13‐DVT‐Deep vein thrombosis	5	1.6	21	2.9	26	80.8 (62.1‐91.5)
G801.00‐Deep vein phleb/thrombophlebitis leg	4	1.3	10	1.4	14	71.4 (45.4‐88.3)
G401.12‐Pulmonary embolus	4	1.3	7	1.0	11	63.6 (35.4‐84.8)
Other	36	11.6	32	4.5	68	47.1 (35.7‐58.8)
**Status at diagnosis**						
Hospital record or death cause	46	14.8	269	37.8	315	85.4 (81.1‐88.9)
Only Referral to specialist	265	85.2	443	62.2	708	62.6 (58.9‐66.1)

^a^ List of most frequent codes, identifying at least 10 possible cases.

## 4 DISCUSSION

We have shown that the PPV of coded VTE entries alone among new users of warfarin/rivaroxaban in THIN primary care database was 32.9%. This increased only slightly to 39.8% when we required cases to also have a coded entry for a referral/hospitalization recorded close in time to the event. However, the PPV increased substantially to almost 70% when the patients' primary care records were manually reviewed enabling scrutiny of other coded entries relating to secondary care referrals, hospital discharge letters, and information originating from death certificates. Furthermore, 30% of cases retained after manual review of the patient's primary care record were not subsequently confirmed as true new cases following scrutiny of the free text comments, showing that this information is required to obtain cases with the highest level of validity. The incidence rate of VTE among our rivaroxaban/warfarin study cohort was overestimated three‐fold when using coded VTE entries alone compared with when using cases confirmed after manual review of the primary care record including the free‐text comments.

Almost half of confirmed VTE cases in our study had no evidence of hospitalization for their event, indicating that there is a high level of outpatient management VTE in UK clinical practice. In the study by Lawrenson et al,^18^ which validated VTE Read code entries in the UK's CPRD among women using a combined oral contraceptive during a 20‐year earlier time period through postal questionnaires sent to PCPs, 20% of confirmed VTE cases did not have a record of hospitalization. These findings could suggest changes to patterns in VTE management in clinical practice over time, that is, increased outpatient management. In a study of 5497 adults with VTE, 95% of PE cases were managed in hospital, while 55% of lower extremity thrombosis cases were treated as outpatients.^19^ Our PPV of almost 70% for hospitalized/referred VTE is lower than the 84% reported by Lawrenson et al,^18^ yet differences in estimated PPVs between studies could also be due to differences in coding and case ascertainment methods. A 71% PPV was reported by Ohman et al^20^ in their validation of International Classification of Diseases (ICD) coded entries for VTE in the National Patient Registry and Death Registry in Sweden.^20^ In the Diet Cancer and Health study, which used data from the Danish Patient Registry, Severinsen et al^21^ reported a 75% PPV for VTE diagnoses coded in hospital wards. In the Cardiovascular Research Network VTE cohort study in the United States, Fang et al^22^ reported a 52% PPV for ICD‐9 VTE codes entered in patient's administrative healthcare records. A 54% PPV was reported by Tuckuviene et al^23^ for VTE diagnoses in the Danish National Patient Registry among patients aged ≤18 years. The confirmation rate in our study varied according to characteristics of patients and of the VTE event. We calculated a 72% PPV for PE and a 68% PPV for DVT, and other studies in adults have also been consistent in finding a higher PPV for PE than DVT.^20^, ^21^, ^22^, ^24^ It is plausible that patients with PE were more likely to be admitted to hospital for observation and treatment. In our study, the PPV among patients hospitalized for their VTE was 85% compared with 63% among those not hospitalized but with a documented referral to a specialist. Other studies have similarly reported notably higher PPVs for VTE among inpatients/emergency admissions than among outpatients.^22^, ^23^, ^24^ We also found a higher PPV among males and among patients aged ≥60 years. This contrasts with findings by Sundbøll et al^24^ who found the PPV for ICD‐coded VTE diagnoses in the Danish National Patient Registry was consistent between the sexes and age groups. Using the same registry, Severinsen et al^21^ found a higher PPV for DVT among men but no difference in the PPV for PE between the sexes and no differences in the PPV for VTE according to age group. In contrast to Sundbøll et al^24^ who reported higher PPVs for first‐time VTE events, in our study, the PPV was higher for recurrent VTE events.

We have previously shown the benefit of accessing and reviewing the data in free‐text comments to validate cases of major gastrointestinal and urogenital bleeding events among our cohort of anticoagulant users, with incidence rates overestimated more than two‐fold when this process was not undertaken.^17^ Studies of other clinical conditions in THIN have similarly highlighted the benefit of manually reviewing patient records, especially with the free text, for case validation.^25^, ^26^, ^27^, ^28^ A strength of our study is the broad study population where inclusion was based on having a first prescription for rivaroxaban or warfarin, and all VTE events whether first time or recurrent were included. The scrutiny of the free‐text comments through manual review not only enabled the acquisition of previously “hidden data” but also avoided missing any crucial information that could happen using an approach involving algorithmic searches in the free text for specific text strings. Furthermore, our operational code lists for DVT/PE in step 1 and for referral/hospitalizations in step 2 were broad in order to maximize the sensitivity of our case identification process.

Manual review of patient records including the free‐text comments is costly and labor intensive, and requires the reviewer to have good medical acumen, yet we have demonstrated its importance in the study of VTE. Our findings are of increasing relevance because of the large number of post‐marketing VTE effectiveness studies currently being carried out among users of NOACs in healthcare databases. Furthermore, databases such as THIN are increasingly being used to generate such real‐world evidence for use in regulatory decision‐making and validation exercises will increase confidence in the use of observational data. If researchers do not have access to the free‐text comments in primary care databases such as THIN in studies of VTE, an acknowledgement of the level of misclassification and resulting bias should be made and taken into consideration when interpreting the results. Additionally, owners of databases such as THIN should be encouraged to make anonymized free‐text comments available for research purposes.

## ETHICS STATEMENT

The study protocol was approved by an Independent Scientific Research Committee for THIN (reference THIN14‐018). No individual patient consent was required because the study used de‐identified data provided by patients as a part of their routine primary care.

## CONFLICT OF INTEREST

LAGR and AR work for CEIFE, which has received research funding from Bayer AG. LAGR has also received honoraria for serving on advisory boards for Bayer AG. GB is a full‐time employee of Bayer AB. PV is a full‐time employee of Bayer AG.

## Supporting information


**Table S1**. Read codes for VTE.

## Data Availability

Data are available from the corresponding author upon reasonable request.
