# Supplementary material for: Validation of venous thromboembolism diagnoses in patients receiving rivaroxaban or warfarin in The Health Improvement Network
Source: Pharmacoepidemiol Drug Saf. 2020 Oct 12;30(2):229–36. doi: 10.1002/pds.5146 (PMC7821274; doi:10.1002/pds.5146)
Supplement: Supplementary file 1 — Table S1. Read codes for VTE. [file PDS-30-229-s001.docx]

**Supplemental table 1.** Read codes for VTE.

| **READ code** | **Description** |
| --- | --- |
| G401.00 | Pulmonary Embolism |
| G401.12 | Pulmonary Embolus |
| G401000 | Post Operative Pulmonary Embolus |
| G401100 | Recurrent Pulmonary Embolism |
| G402.00 | Pulmonary Infarct |
| G80..00 | Phlebitis And Thrombophlebitis |
| G800.12 | Saphenous Vein Thrombophlebitis |
| G800300 | Thrombophlebitis Of The Long Saphenous Vein |
| G800400 | Thrombophlebitis Of The Short Saphenous Vein |
| G801.00 | Deep Vein Phlebitis And Thrombophlebitis Of The Leg |
| G801.11 | Deep Vein Thrombosis |
| G801.12 | Deep Vein Thrombosis, Leg |
| G801.13 | Dvt - Deep Vein Thrombosis |
| G801500 | Deep Vein Phlebitis Of The Leg Unspecified |
| G801600 | Thrombophlebitis Of The Femoral Vein |
| G801700 | Thrombophlebitis Of The Popliteal Vein |
| G801800 | Thrombophlebitis Of The Anterior Tibial Vein |
| G801900 | Thrombophlebitis Of The Dorsalis Pedis Vein |
| G801a00 | Thrombophlebitis Of The Posterior Tibial Vein |
| G801b00 | Deep Vein Thrombophlebitis Of The Leg Unspecified |
| G801c00 | Deep Vein Thrombosis Of Leg Related To Air Travel |
| G801d00 | Deep Vein Thrombosis Of Lower Limb |
| G801e00 | Deep Vein Thrombosis Of Leg Related To Intravenous Drug Use |
| G801f00 | Deep Vein Thrombosis Of Peroneal Vein |
| G801g00 | Recurrent Deep Vein Thrombosis |
| G801h00 | Unprovoked Deep Vein Thrombosis |
| G801j00 | Provoked Deep Vein Thrombosis |
| G801z00 | Deep Vein Phlebitis And Thrombophlebitis Of The Leg Nos |
| G802.00 | Phlebitis And Thrombophlebitis Of The Leg Nos |
| G802000 | Thrombosis Of Vein Of Leg |
| G80y.00 | Other Phlebitis And Thrombophlebitis |
| G80y.11 | Phlebitis And/Or Thrombophlebitis Of Iliac Vein |
| G80y400 | Thrombophlebitis Of The Common Iliac Vein |
| G80y500 | Thrombophlebitis Of The Internal Iliac Vein |
| G80y600 | Thrombophlebitis Of The External Iliac Vein |
| G80y700 | Thrombophlebitis Of The Iliac Vein Unspecified |
| G80y800 | Phlebitis And Thrombophlebitis Of The Iliac Vein Nos |
| G80yz00 | Other Phlebitis And Thrombophlebitis Nos |
| G80z.00 | Phlebitis And Thrombophlebitis Nos |
| G80z100 | Thrombophlebitis Nos |
| G80zz00 | Phlebitis And Thrombophlebitis Nos |
| G82..00 | Other Venous Embolism And Thrombosis |
| L096400 | Pulmonary Embolism Following Abortive Pregnancy |
| L413.00- L413z00 | Antenatal Deep Vein Thrombosis |
| L414.00 | Postnatal Deep Vein Thrombosis |
| L414.11 | Dvt - Deep Venous Thrombosis, Postnatal |
| L414.12 | Phlegmasia Alba Dolens - Obstetric |
| L43..00- L43zz00 | Obstetric Pulmonary Embolism |
| Sp12200 | Post Operative Deep Vein Thrombosis |
| Zv12800 | [V] Personal History Deep Vein Thrombosis |
| Zv12811 | [V] Personal History Dvt- Deep Vein Thrombosis |
| Zv12900 | [V] Personal History Of Pulmonary Embolism |
| 14a8.12 | H/O: Thrombosis |
| 14a8100 | H/O: Deep Vein Thrombosis |

ICD-10 cross-matched codes

Pulmonary embolism (I26;I260;I269)

Venous thrombosis and thrombophebiltis (I80; I800; I801; I802; I803; I808; I809; I82;O223;O871)
